# ACM and rectangular images: Overlapping partitions, implementation, and periodicity analysis

**DOI:** 10.1371/journal.pone.0303589

**Published:** 2024-08-12

**Authors:** Anthony O’Dea

**Affiliations:** Chemical Engineering Department, University of California, Santa Barbara, Santa Barbara, CA, United States of America; Deakin University, AUSTRALIA

## Abstract

The Arnold Cat Map (ACM) is a popular chaotic map used in image encryption. Chaotic maps are known for their sensitivity to initial conditions and their ability to permute, or rearrange, pixels. However, ACM is periodic, and its period is relatively short. This periodicity decreases the effective key-space and security of a cryptosystem using ACM. Further, ACM is typically only able to be performed on square images. To solve the low periodicity and typical limitation to square images, this paper proposes performing ACM on overlapping square partitions which cover the entirety of an image. The presence of overlap results in a greatly increased image period. The resulting system will be referred to as overlapping ACM or OACM. Several papers have already discussed systems involving overlapping ACM. However, they did not discuss the implementation or periodicity of such a system in detail. This paper does cover the implementation and periodicity analysis of OACM and proposes a simple symmetric encryption system which uses OACM. The proposed encryption system is not as sophisticated or secure as other modern encryption schemes, since it is mainly intended as an initial test of OACM’s utility. Histogram and sensitivity analyses did however indicate a level of security against various cryptographic attacks, and OACM performed reasonably in both the permutation and diffusion stages of the cryptosystem.

## Introduction

With a rise in the use of cloud storage, electronic communications, and electronic health records, image encryption as a means to protect privacy is more important than ever. Without modification, traditional encryption techniques are not adequate for image encryption. This difficulty principally arises from normal images’ bulk data capacity and the high correlation between adjacent pixel values. Regardless, strong image encryption techniques rely on the same framework of frustrating statistical analysis as traditional techniques. This framework being diffusion and confusion, which was described by Claude Shannon in 1945 [1].

Diffusion refers to the dissipation of meaningful statistical patterns within the plaintext during the encryption process. This means that any statistical patterns present within the ciphertext should not be relevant to the plaintext. For image encryption, this typically means that the probability of any given color value at a position should appear to be random. For cipher images that fulfill this property of diffusion, their color value histograms will be flat, and there should be no correlation between the color values of adjacent pixels. Diffusion also refers to the sensitivity of the ciphertext to changes in the plaintext. Confusion refers to the relationship between the key and the ciphertext, which should be a very complex relationship such that any given value in the ciphertext depends on multiple parameters from the key.

A high sensitivity to the plain image can be achieved by generating a hash from the image, using algorithms such as MD5 and SHA 256 [2, 3]. The hash value is then used to generate parameters for the encryption process. In other words, the hash algorithm generates an image specific key. This is typically used in addition to a user-specified/fixed key. Both of these keys must be known for decryption. The dependency of the ciphertext on the plaintext as a result of these hash parameters increases a cryptosystem’s resistance towards known and chosen plaintext attacks [3].

A common image encryption architecture is permutation-diffusion, which is a two stage process of permutation followed by diffusion [4]. Permutation typically refers to the rearrangement or shuffling of pixels within an image. Permutation can also refer to the rearrangement of bits [5–11]. The permutation phase in permutation-diffusion is often associated with chaotic maps, but can also be performed with scan patterns, jigsaw transform, and other methods [12, 13]. During the diffusion stage, the color values are changed, and the diffusion principle of encryption is more fully achieved. Note that an encryption scheme that only involves permutation will always be weak to plaintext attacks [14].

The Arnold cat map is an area preserving linear transformation which is commonly used in image encryption schemes. ACM is one of several chaotic maps used in image encryption [15]. ACM is mainly used to rearrange an image’s pixels such that after some number of iterations, the resulting image will appear to have been shuffled randomly. ACM can also be used to alter an image’s color values. ACM in matrix form is:

$$\begin{array}{c} {}[\begin{array}{c} x^{\prime }\\ y^{\prime } \end{array}]=[\begin{array}{cc} 1 & 1\\ 1 & 2 \end{array}][\begin{array}{c} x\\ y \end{array}]\;\text{mod}\;N=[\begin{array}{c} A \end{array}][\begin{array}{c} x\\ y \end{array}]\;\text{mod}\;N \end{array}$$ (1)

[A] is shorthand for the ACM matrix. x’ and y’ indicate the new location of the pixel formerly at x and y. N is the side length in pixels of a square image. This paper will use the terms width and height to refer to the horizontal and vertical lengths of non-square images when necessary. For decryption, the inverse of the ACM matrix can be used to move pixels back to their original state.

**Fig 1:**
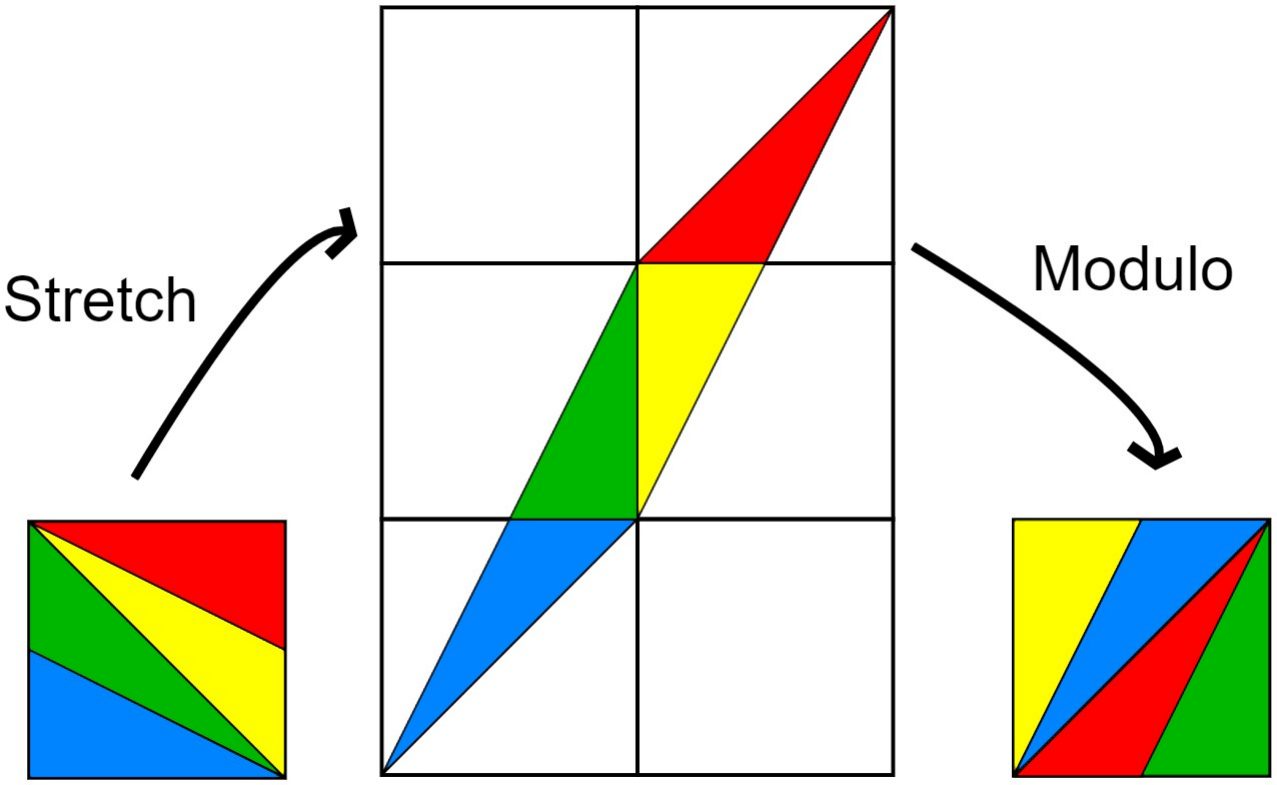
Standard ACM operation. The ACM matrix stretches the image while retaining the number of pixels, and the modulo operation cuts the stretched image into pieces and places these pieces back into the image bounds.

The standard ACM transformation can be visualized in Fig 1.

ACM is periodic, which means that after some number of iterations, the system will return to its original state. The period, P(N), is a function of N and certain relations have been found between N and P(N):

$$\begin{array}{cc} P(N) & =3N\;for\;N=2*5^{k}\\ P(N) & =2N\;for\;N=5^{k}\;or\;6*5^{k}\\ P(N) & <=\frac{12}{7}N\;for\;other\;N \end{array}$$

With k as positive integers. The period never exceeds 3N [16]. For common image sizes, the period is considerably smaller than this maximum, as shown in Table 1.

**Table 1:** Regular ACM data.

Size	256	512	1024	1080	2048	2992	3000	4320
Period	192	384	768	180	1536	180	1500	360

Periodicity limits the effective key-space and increases susceptibility of the cryptosystem to brute force attacks. Cryptosystems involving chaotic maps with weak key-spaces have been broken before [17].

A pixel within an image undergoing ACM has its own period, which is the minimum number of iterations before returning to its original location. The path a pixel takes during these iterations is referred to as its orbit. All of the pixels along the same orbit have the same period, which is equal to the number of pixels in that orbit. The (0, 0) pixel does not move, so it has a period of 1. The overall P(N) of an image will be the least common multiple of the individual pixel periods. Excluding the origin’s orbit, orbits in images with prime N (except for N = 5) will all have the same length [18].

**Fig 2:**
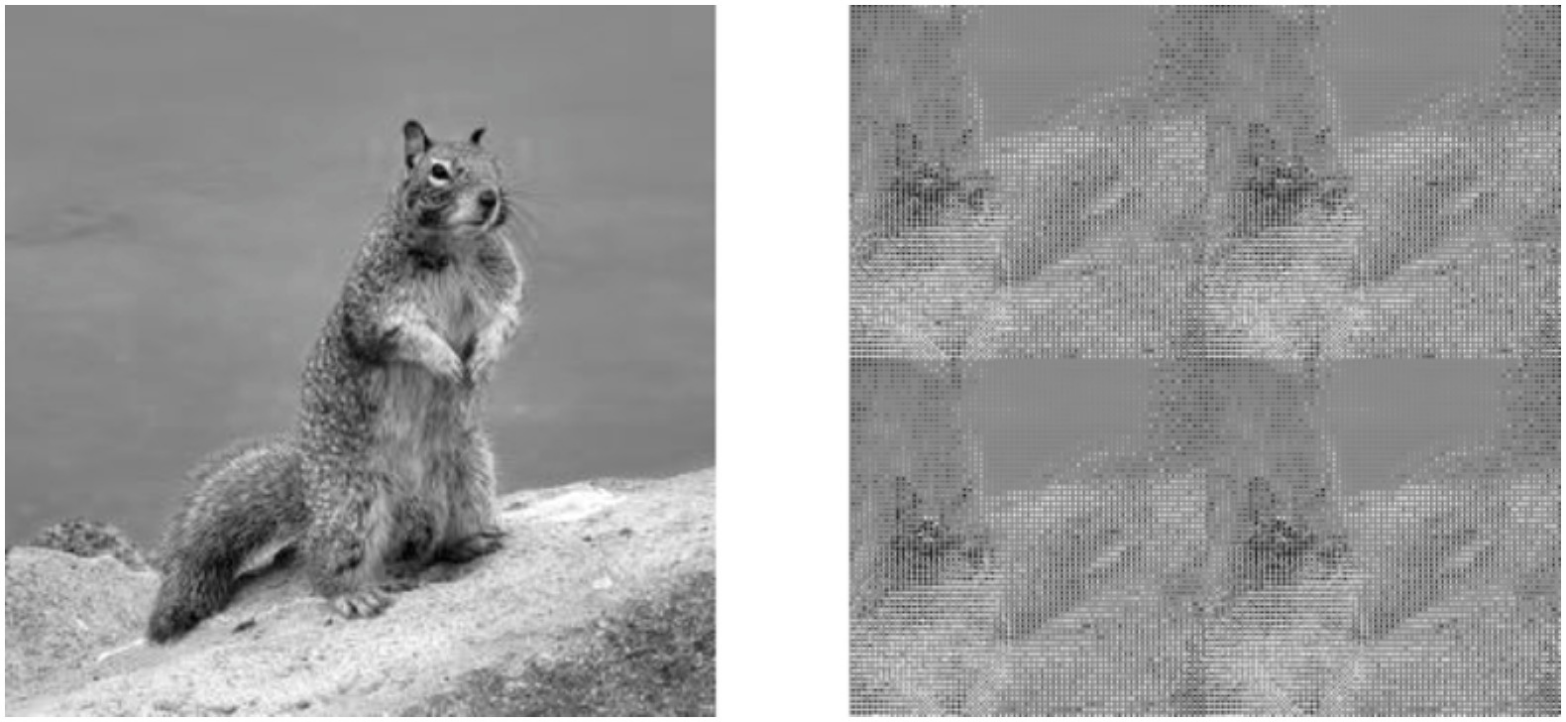
256 X 256 squirrel image vs after P(256)/2 iterations. The original image is somewhat evident at P(256)/2 iterations.

**Fig 3:**
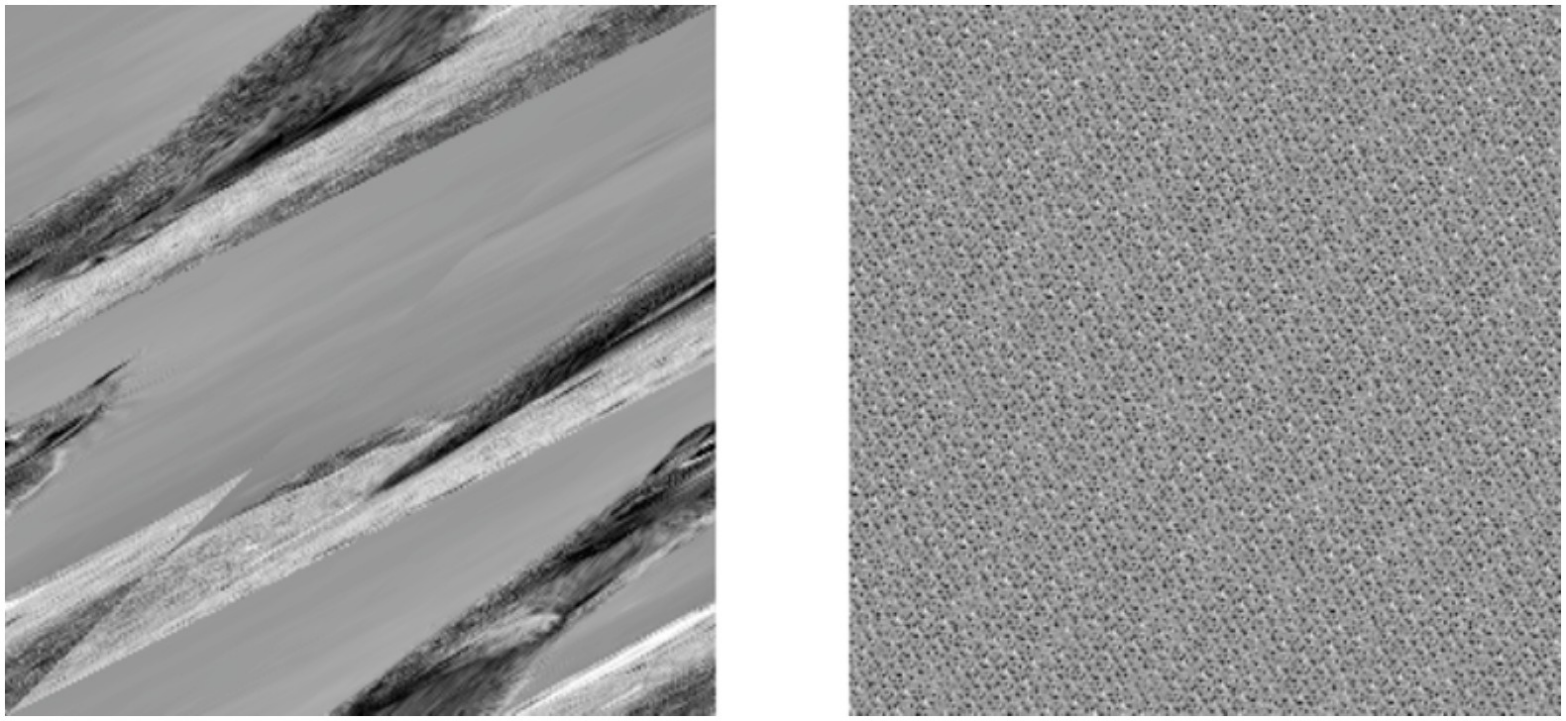
256 X 256 squirrel image after one iteration vs ten. The image is still somewhat recognizable after one iteration, but is more like noise after only ten iterations.

At certain iterations of ACM before P(N), the original image may reappear in some manner [19]. This effect may be seen in Fig 2. These iterations will be referred to as ghost periods and typically are simple fractions of P(N). Although not typically discussed, these ghost periods do decrease the security of ACM. The typical randomization process across iterations is shown in Fig 3 for comparison.

### Implementing ACM

To implement ACM programmatically, Eq 1 may be rewritten as:

$$\begin{array}{c} x^{\prime }=\left(x+y\right)\;\text{mod}\;N,\;y^{\prime }=\left(x+2y\right)\;\text{mod}\;N \end{array}$$ (2)

ACM is performed some number of iterations, Z, on an image during the encryption process. Rather than repeatedly performing ACM Z times, all of those iterations can be replaced with a single operation. This can be done in several ways. Multiple iterations of Arnold cat map may be represented via:

$$\begin{array}{c} {}[\begin{array}{c} x^{Z}\\ y^{Z} \end{array}]=[\begin{array}{c} A \end{array}{]}^{Z}[\begin{array}{c} x\\ y \end{array}]\;\text{mod}\;N \end{array}$$ (3)

Where [*A*]^*Z*^ is simply the ACM matrix raised to the power Z. However, the values in [*A*]^*Z*^ can quickly become large enough to cause numerical errors, unless otherwise accounted for. To avoid these large values, [*A*]^*Z*^ can be replaced with *A*_*Z*_, which can be found with the following iterative sequence:

$$\begin{array}{c} {}[\begin{array}{c} A_{j} \end{array}]=[\begin{array}{c} A_{j-1} \end{array}][\begin{array}{c} A \end{array}]\;\text{mod}\;N \end{array}$$ (4)

The orbits of the ACM operation can also be used to jump directly to any given iteration. Let’s define an orbit as a list of n pixels with indices from 0 to n—1. A single iteration of ACM will result in a rightward shift along this list by 1 index, with the rightmost pixel moving to index 0. With *i*(*p*) as the index of pixel p, the location of a pixel after Z iterations can be represented as:

$$\begin{array}{c} i(p)=(i(p)+Z)\;mod\;n \end{array}$$ (5)

Permuting every pixel in every orbit according to this operation will result in the same image as performing ACM Z times. Like the above methods, as Z gets larger, it will take considerably less time than performing each ACM operation individually. This orbit based permutation method will work for permutations besides ACM as well.

### Modifications to ACM

Several modifications to ACM have been proposed in literature. These modifications are primarily used to increase the key-space and the period. The simplest of these modifications involve replacing elements of the matrix with integer variables. ACM can also be extended to 3D [20, 21]. Nonlinear perturbations can also be added to ACM, which can considerably increase the period [22]. Linear displacements can also be added onto ACM to change the effective origin coordinate [23].

One of the common generalization of ACM is:

$$\begin{array}{c} {}[\begin{array}{c} x^{\prime }\\ y^{\prime } \end{array}]=[\begin{array}{cc} 1 & P\\ Q & 1+PQ \end{array}][\begin{array}{c} x\\ y \end{array}]\;\text{mod}\;N \end{array}$$ (6)

Where P and Q are integers ranging from 0 to N—1. The values of P and Q can impact the period, and this system has been studied thoroughly [24, 25]. The determinant will always be 1 for this matrix, so the transformation is area preserving. A matrix will be area preserving if its determinant is ±1. This property of area preservation is a commonly mentioned feature of ACM and its extensions [18, 21, 26]. However, this is not actually necessary for a transformation and modulo operation to be usable for image encryption. What matters is that the operation is invertible, as in it can be reversed. When a matrix transformation and modulo operation is not invertible, what occurs is that 2 or more points from the plain image are mapped to the same location in the cipher image. This destroys information, and prevents the operation from being reversed. The transformation and modulo operation of a square image will be invertible if *gcd*(*det*[*A*], *N*) = 1 [27]. This will always be true for area preserving matrices, but also allows for matrices with determinants besides ±1.

A number of papers discuss transformation matrices where every matrix element is a variable [26, 27]. Importantly, such a system has been described as able to work on non-square images when a specific set of conditions are met [28, 29]. This operation is called the 2D rectangular transform and is defined as 7:

$$\begin{array}{c} \left[\begin{array}{c} x^{\prime }\\ y^{\prime } \end{array}]=[\begin{array}{cc} a & b\\ c & d \end{array}][\begin{array}{c} x\\ y \end{array}]\;\text{mod}\;[\begin{array}{c} W\\ H \end{array}\right] \end{array}$$ (7)

The modulo operation has been modified to account for when the height and width are different from each other. More critically, is the understanding of when this operation can be performed reversibly on non-square images. This is possible when 8:

$$\begin{array}{c} \begin{array}{c} \left\{ \begin{array}{c} gcd(det[A],p)=1\\ gcd\left(a,p_{W}\right)=1\;\;and\;\;gcd\left(d,p_{H}\right)=1\\ \left(b\;mod\;p_{W}\right)=0\;\;or\;\;\left(c\;mod\;p_{H}\right)=0 \end{array}\right.\\ with:\;p=gcd\left(W,H\right),\;p_{W}=W/p,\;p_{H}=H/p \end{array} \end{array}$$ (8)

So under those conditions, ACM and other transformation matrices can be used to successfully encrypt and decrypt non-square images. This is very important, but is under-discussed in literature.

As opposed to a modification to the actual ACM operation, like above, this paper will propose and analyze a means of applying ACM to an image. This application method bypasses the above image size constraints and has much greater periodicity than traditional ACM.

## Overlapping ACM

Previous works have discussed the use of overlapping partitions for ACM and other techniques, but they did not focus on the periodicity or implementation of such systems [30–33]. Overlapping partitions can extend ACM to non-square images and will also greatly increase the overall image period. This paper proposes a means of performing ACM on overlapping square blocks in a pattern similar to a regular grid. While this will limit the possible number of permutations

There are several key parameters to this problem: image height and width, square size, amount of overlap, and number of squares. The number of squares is determined by the other parameters and can not be directly set within the proposed method detailed below.

The method begins by producing a list of x and y coordinates for the upper left corner of each square, based on the given parameters. The respective tiling will be made with the least number of squares possible. The specified amount of overlap must be broken in cases where the overlapping squares would not otherwise properly cover the entire image. The overlap in these cases will be referred to as geometric overlap, as opposed to specified overlap. The coordinates are found via Algorithm 1.

**Algorithm 1** Square Locations

for y from 0 to image height—square size—1 by 1’s

 if (y-1) Mod (square size—overlap) = 0

  add y to y list

 end

end

add height-squareSize to y list

for x from 0 to image width—square size—1 by 1’s

 if (x-1) Mod (square size—overlap) = 0

  add x to x list

 end

end

add width-squareSize to x list

ACM is then performed in order on each square specified by those two lists. The set of those ACM operations make up a single operation of OACM. Decryption is performed via inverse ACM on each square in the reverse order.

The total number of possible tilings which can be produced by algorithm 1 is limited by the smallest image dimension. The square partition size can range from 2 to the limiting dimension. Overlap can range from 1 to square size minus 1. Overlap must also be ≥ 2*square size—limiting dimension. A specified overlap which breaks that rule will be forced to equal a geometric overlap. Table 2 suggests a second order relationship with the limiting dimension, N.

**Table 2:** Possible number of tilings.

Limiting Dimension (N)	512	1024	2048	3000
Possible Tilings	65280	261632	1047552	2248500

**Fig 4:**
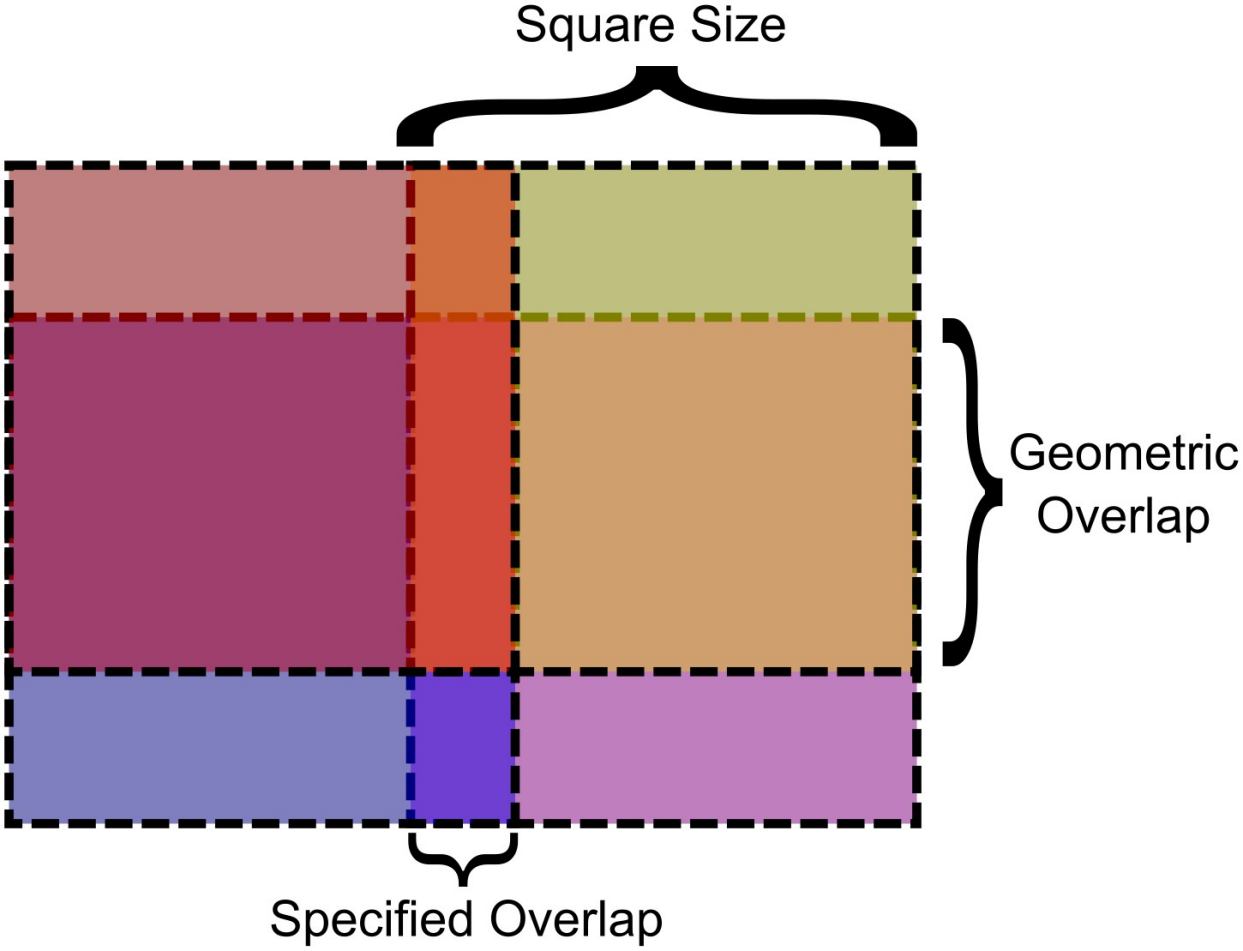
Diagram of OACM on a rectangular image using 4 semi-transparent colored squares.

**Fig 5:**
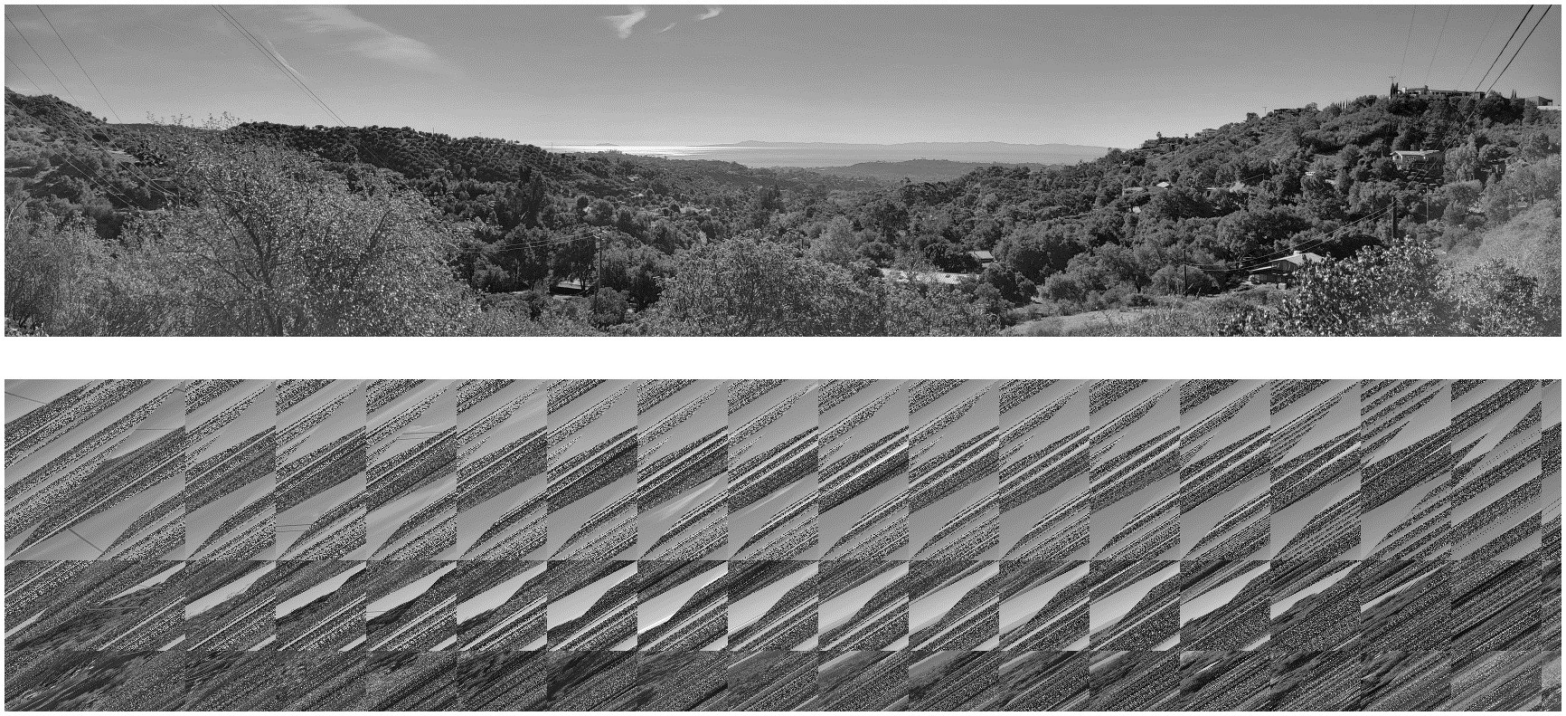
1722 by 367 panorama. Permuted once by OACM with square size as 200 and overlap as 100.

Fig 4 portrays a simple OACM system with 4 squares overlaying a rectangular image. The geometric overlap is larger than the specified overlap, which will always be true for systems that require geometric overlap. A smaller overlap would extend the squares past the bounds of the image. Fig 5 also helps visualize how OACM works.

### Implementing overlapping ACM

Unlike ACM, multiple iterations of overlapping ACM (OACM) can not be performed with a single matrix multiplication. However, Eq 5 is still applicable and can be used to greatly decrease computation time. The orbits used in the implementation of Eq 5 can all be calculated from a single operation of OACM.

**Fig 6:**
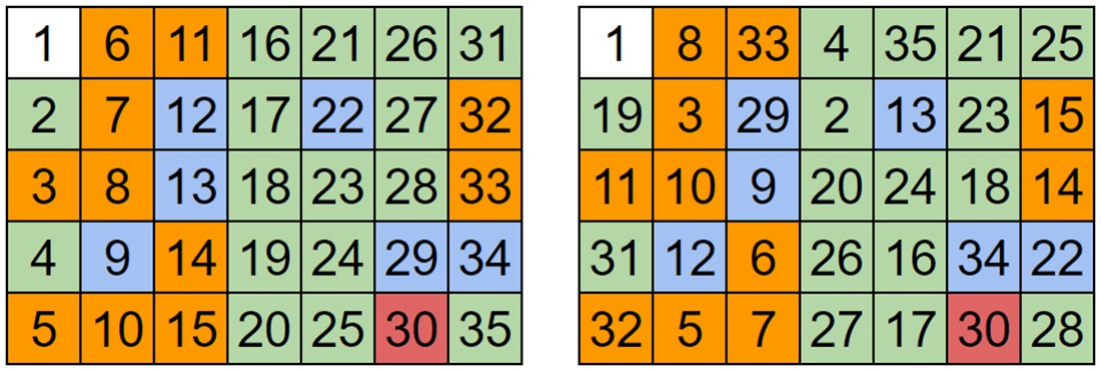
Matrix of indices (left) vs after a single permutation of OACM (right) with square size as 4, overlap as 2, and P and Q both as 1. The colors represent pixels within the same orbit.

**Fig 7:**
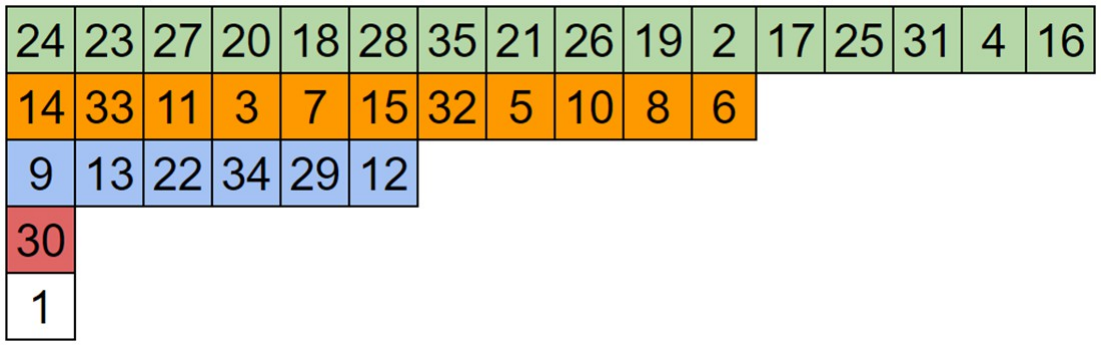
The 5 orbits of the OACM system shown in Fig 6. Pixel 30 is stationary, in addition to the origin. These orbits can then be used to calculate the location of a pixel after any number of iterations. The lengths of the orbits can also be used to quickly determine the period of the system.

An index matrix is a matrix where the value at an index is equal to the index. OACM is performed on an index matrix. Now, at any given index is a value representing the index of a pixel that is moving along the same orbit as the original index. Moving to that second indexes’ position within the permuted index matrix yields another pixel in the same orbit, and so on. Figs 6 and 7 represent a small example using the above technique.

## Periodicity analysis

A limiting/simplifying case of OACM is when a rectangle is to be tiled by the minimum number of squares with size equalling the image height. For rectangles with aspect ratio W:H between 1:1 and 2:1, this tiling is simply one square on the leftmost and rightmost sides. For squares, a simplifying case would be a tiling with four squares in each corner with edge lengths > half the image size.

Tables 3 and 4 indicate the overall period for simplified rectangular and square OACM systems respectively. The periods within these tables were found as the least common multiple of the orbit lengths.

**Table 3:** Period data for rectangles tiled with two squares of size H.

W:H	W	H	Period
16:9	1920	1080	9.2 * 10^489^
3:2	1620	1080	1.7 * 10^77^
4:3	1280	960	1.3 * 10^332^
16:9	1280	720	6.6 * 10^344^
3:2	1080	720	4.0 * 10^37^
4:3	320	240	1.9 * 10^81^

**Table 4:** Period data for square images tiled with 4 overlapping squares.

Image Size	Square Size	Period
1080	720	1.6 * 10^163^
1024	768	2.1 * 10^457^
1024	640	4.3 * 10^486^
768	512	3.3 * 10^277^
512	384	8.0 * 10^227^
256	192	4.4 * 10^117^

**Fig 8:**
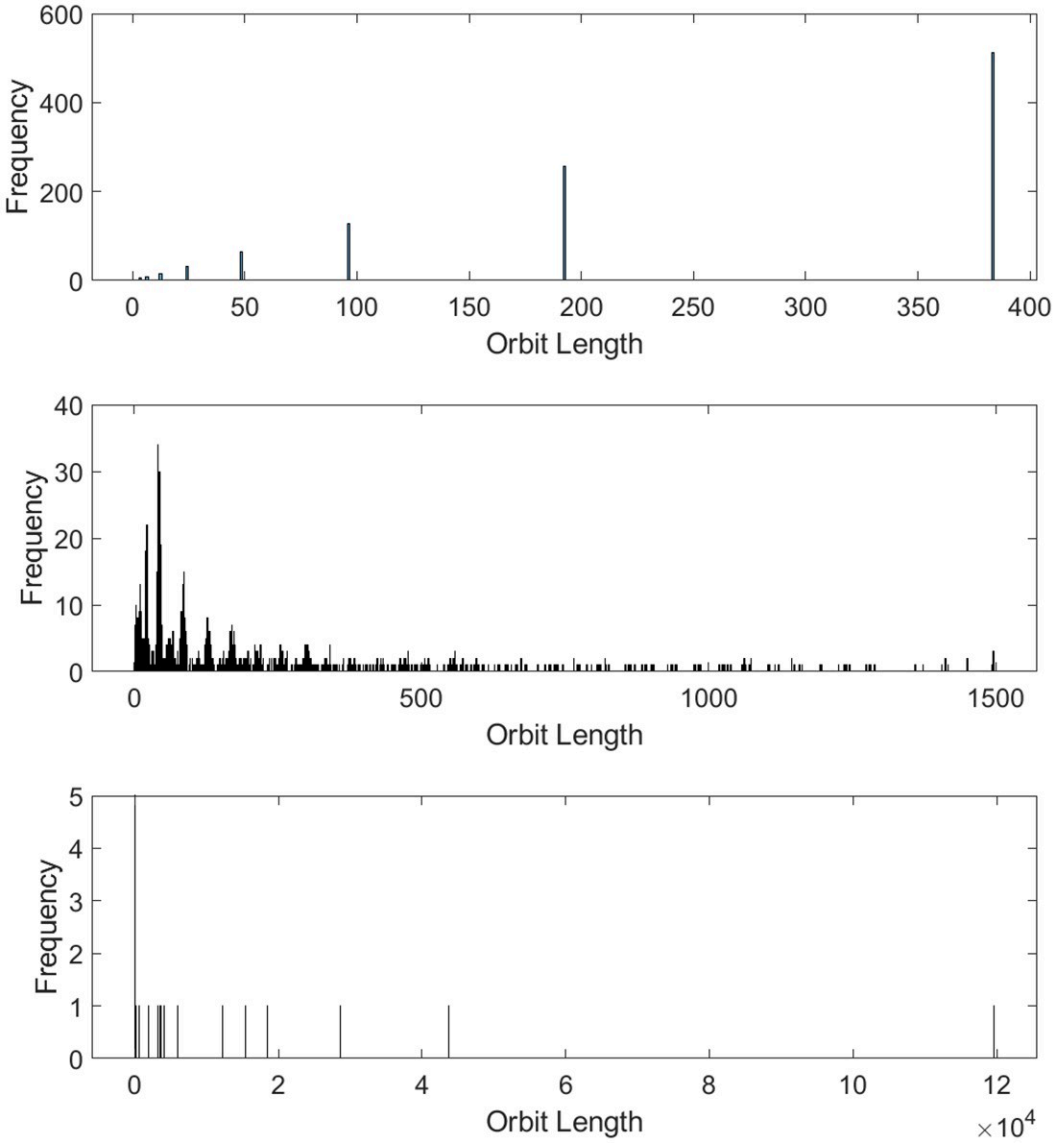
Histograms indicating the frequency of orbit lengths in 512 by 512 images. From top to bottom, regular ACM, OACM with 4 squares of size 384, and OACM with squares of size 100 and an overlap of 25.

The large increases in image periodicity compared to regular ACM are a direct result of increases in the lengths of orbits and the number of unique orbit lengths. The orbit length distributions of normal ACM and two OACM systems are shown in Fig 8. Note that certain combinations of square size and overlap can result in very long orbit lengths. This limits the effective period to the length of that orbit. This effective period will be referred to as a ghost period, even though it is somewhat different from regular ACM’s ghost periods.

**Fig 9:**
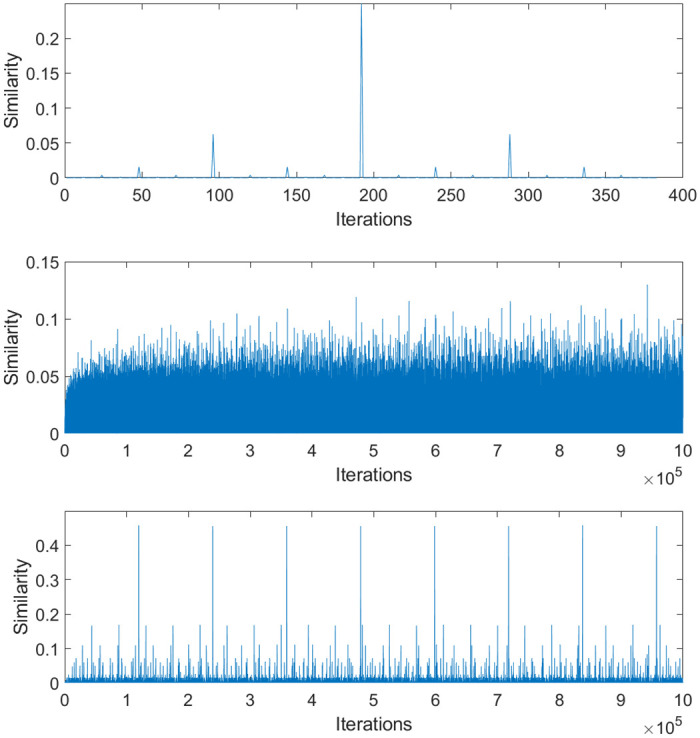
Similarity graphs for 512 by 512 images. From top to bottom, regular ACM, OACM with 4 squares of size 384, OACM with squares of size 100 and an overlap of 25. Note the presence of peaks around 50% similarity in the bottom graph, which comes from a single large orbit of length 119657.

**Fig 10:**
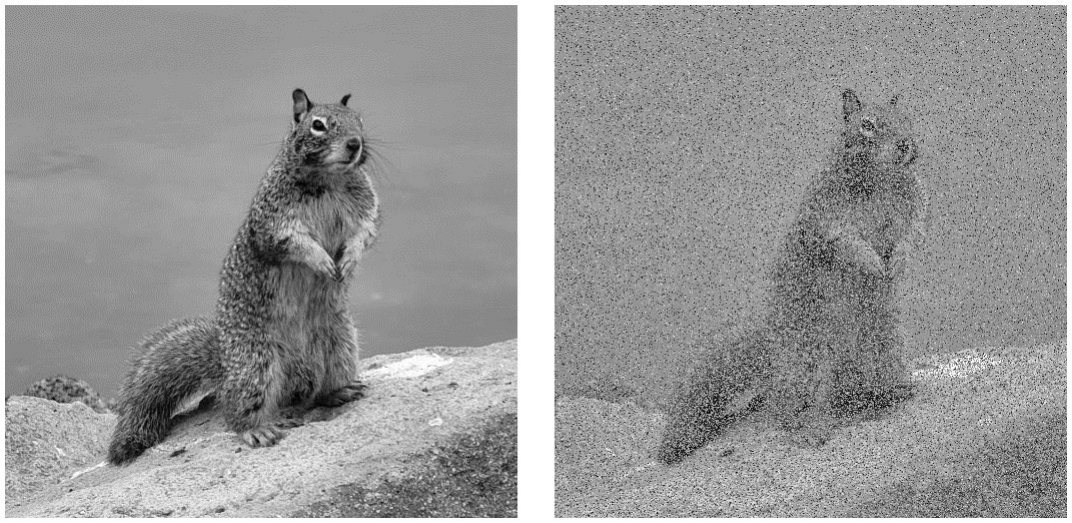
512 X 512 squirrel image vs after 119657 iterations of OACM with square size as 100 and overlap as 25. The singular orbit of length 119657 having returned to its original state makes the image easily recognizable.

Similarity graphs like those in Fig 9 can also be used to detect ghost periods. Similarity graphs represent the number or percentage of pixels back in their original location vs the number of iterations. Noticeable peaks in these graphs will occur when multiple orbits return to their original state at the same time or when an orbit of sufficient length returns to its original state. The impact this can have on an image is shown in Fig 10. Unless accounted for, these ghost periods will decrease the effective key-space and security of a system which uses OACM.

Given that OACM provides some level of control over the orbit lengths, what set of these lengths will yield the maximum possible image period? A similar question was posed by Edmund Landau around the start of the 20th century [34]: what partitions of n elements will provide the maximal order? The LCM of the lengths of the partitions is the order, and would also be maximized. The “Landau series,” s(n), for n elements will be a set of partition lengths which provide maximal order. There may be multiple s(n) for the same n. The Landau function, g(n), provides the order for s(n). Note that the Landau function is not a singular complete function, and there are several existing methods for finding g(n) with varying speeds and ranges of accuracy [35, 36]. The prime factorization of g(n) may be used to find s(n) where any recurring primes within the prime factor list are replaced by their product.

The values of g(n), with n as the number of pixels, provide an upper bound for OACM’s and similar systems’ periodicity. For example, a 512 by 512 image with Landau sized partitions would have a period of g(262144), equalling 4.3 * 10^826^. Note the much smaller OACM period values in Tables 3 and 4.

## Proposed encryption scheme

**Fig 11:**
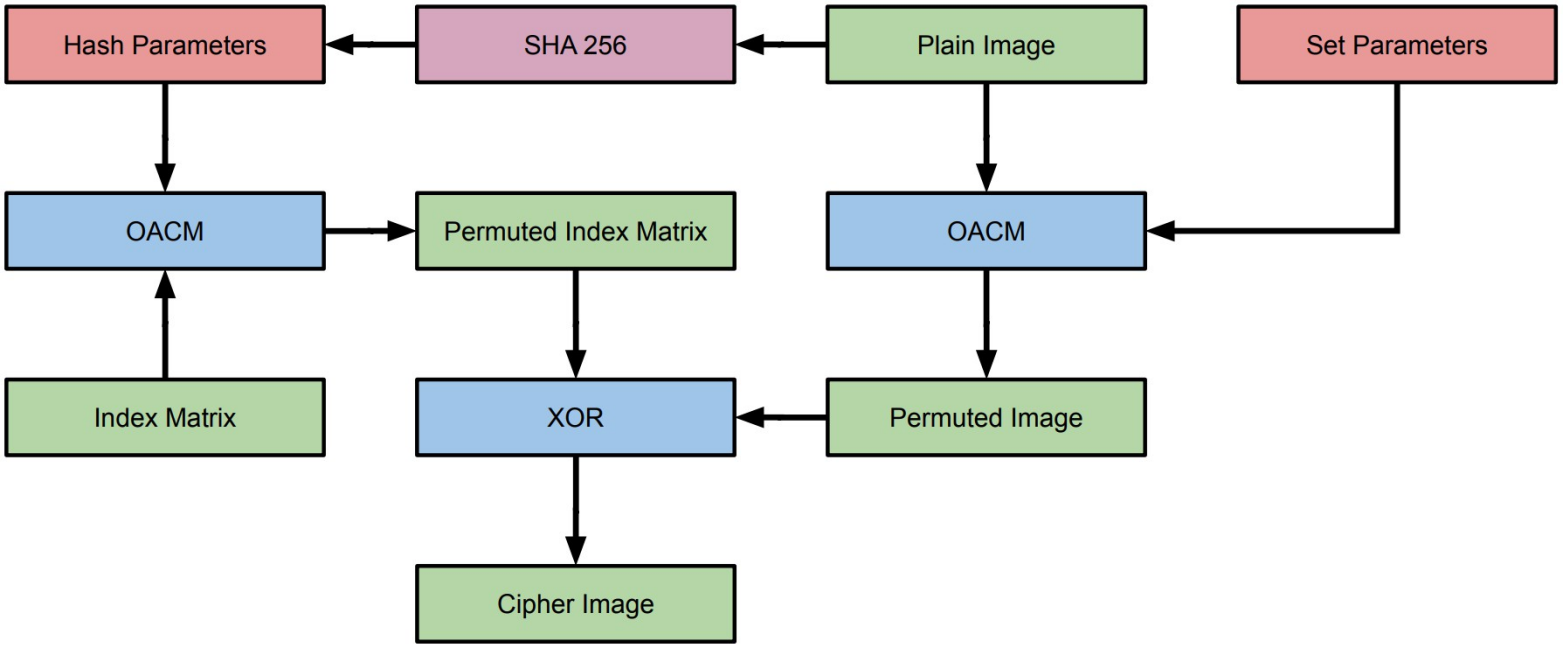
Proposed encryption scheme. This is a pared down permutation-diffusion scheme. Typically, there would be multiple rounds of these operations.

This paper proposes using OACM in the scheme shown in Fig 11. The set parameters are used to determine the OACM permutation performed on the plain image. Hash parameters derived from the SHA 256 hash of the plain image will be used to permute an index matrix with the same dimensions as the plain image. The permuted index matrix is then XORed with the permuted plain image to yield the cipher image. Decryption will involve performing the XOR operation again, and then performing reverse OACM on the resulting matrix to yield the plain image. The individual ACM operations used in this process will be the common two parameter extension of ACM. This adds P and Q as parameters. Size, overlap, and the number of iterations will be the other parameters.

### Hash key

The SHA 256 hash provides 256 bits from which the hash parameters can be derived. Fractions *F*_*S*_, *F*_*P*_, *F*_*Q*_, and *F*_*O*_ are each generated from the XOR of 4 sets of 10 bits from the hash. The resulting binary sequence from each XOR operation is converted to an integer between 0 and 1023, and then converted to a fraction between 0 and 1. These fractions are used to generate values for square size, P, Q, and overlap. This parameter scheme considerably under-utilizes the 256 bit hash. For Eq 9, length refers to the smaller image dimension and limit will account for the geometric constraints placed on overlap.

$$\begin{array}{c} \left\{ \begin{array}{c} F_{S}=int\left(H_{1}^{10}⊕H_{11}^{20}⊕H_{21}^{30}⊕H_{31}^{40}\right)/1023\\ F_{P}=int\left(H_{41}^{50}⊕H_{51}^{60}⊕H_{61}^{70}⊕H_{71}^{80}\right)/1023\\ F_{Q}=int\left(H_{81}^{90}⊕H_{91}^{100}⊕H_{101}^{110}⊕H_{111}^{120}\right)/1023\\ F_{O}=int\left(H_{121}^{130}⊕H_{131}^{140}⊕H_{141}^{150}⊕H_{151}^{160}\right)/1023\\ Size=\left(Length-10\right)*F_{S}+10\\ P=\left(Size-1\right)*F_{P}+1\\ Q=\left(Size-1\right)*F_{Q}+1\\ Overlap=\left(Size-limit\right)*F_{O}+limit-1\\ Iterations=int(H_{161}^{208}⊕H_{209}^{256}) \end{array}\right. \end{array}$$ (9)

### Results

This section focuses on the encryption of a 384 by 256 rabbit image. The parameters for its encryption are shown in Table 5. As shown in the table, a single bit change in the plain image can result in a considerable change to the hash parameters. Sensitivity towards the plain image helps protect against differential attacks.

**Table 5:** Parameters from the rabbit image. The origin coordinate of the plain image was increased by 1 before the image was run through SHA 256 again to determine the Bit-Off hash parameters.

	Iterations	Square Size	Overlap	P	Q
Set Parameters	1000	50	25	1	1
Hash Parameters	113913172259407	110	18	4	20
Bit-Off Hash Parameters	144443707499487	138	73	98	125

**Fig 12:**
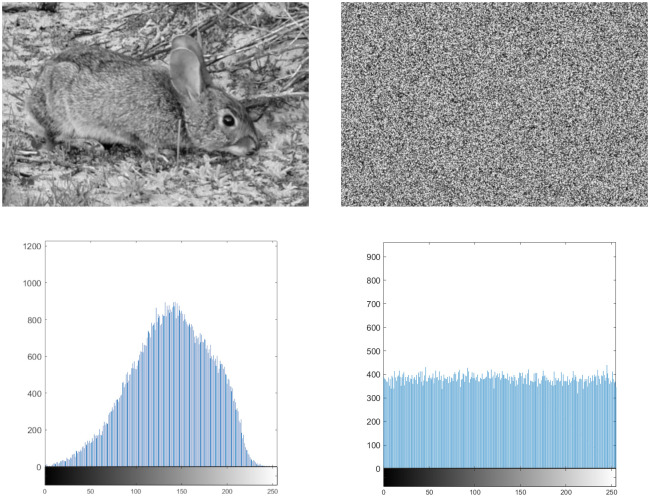
256 by 384 rabbit plain vs cipher image.

The level of randomness in the cipher image can be seen quite clearly in Fig 12. The cipher image histogram is uniform, which prevents any useful information about the plain image from being obtained. Note that the change in the image’s histogram comes solely from the XOR operation during diffusion. The permutation does not change the histogram.

**Fig 13:**
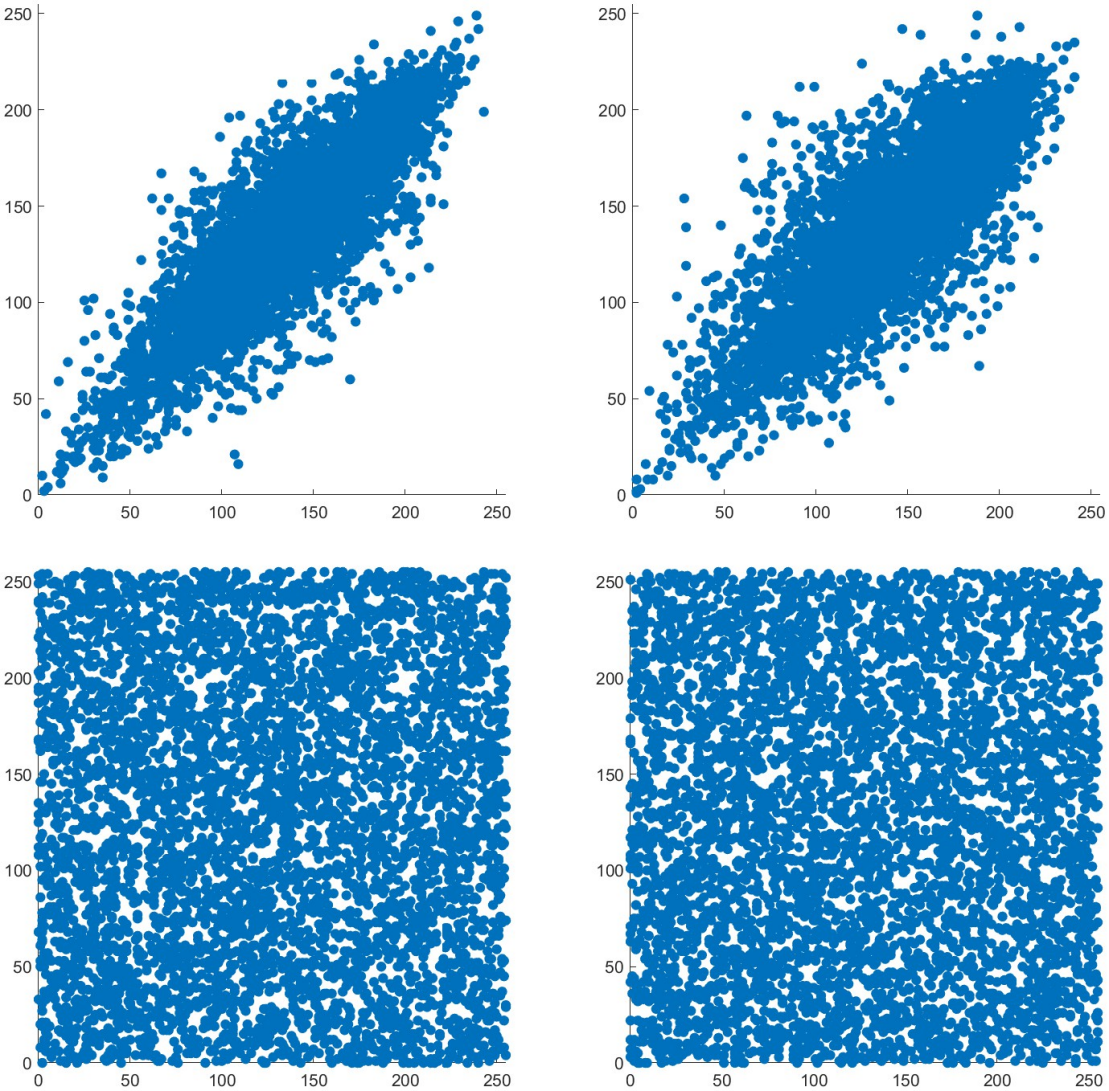
Horizontal (left) and vertical relationships between 5000 random pixels in the plain rabbit (top) and cipher rabbit.

The correlation between adjacent pixels is expected to greatly decrease upon encryption. This can be visualized with correlation plots as in Fig 13 or quantified with correlation coefficients as in Table 6. The horizontal and vertical correlation coefficients for the cipher images are all around zero, while the plain image coefficients are closer to one. This lack of correlation also indicates resistance against statistical attacks.

**Table 6:** Correlation coefficients between adjacent pixel values. NPCR and UACI given a single bit change in the plain image.

	Rabbit	Squirrel	Mandrill	Camera-man	All Black
Plain Horizontal CC	0.8996	0.9757	0.9072	0.9696	NA
Plain Vertical CC	0.8757	0.9699	0.8637	0.9702	NA
Cipher Horizontal CC	0.0017	0.0123	0.0032	-0.0083	-0.0003
Cipher Vertical CC	0.0030	0.0136	0.0013	0.0052	0.0036
NPCR	0.9959	0.9961	0.9962	0.9961	0.9964
UACI	0.3338	0.3339	0.3347	0.3348	0.3368

Table 6 also includes the Number of Pixels Change Rate (NPCR) and Unified Average Changing Intensity (UACI) when there is a single bit change in the plain image. NPCR and UACI are often used to evaluate the sensitivity of a cryptosystem to changes of specific parameters and the plain image. Sensitivity to the plain image indicates security against differential attacks. NPCR compares two images of the same size and represents the rate at which two pixels in the same location have different values. The images may be two different cipher images with a small change to the key. They could also be the plain image vs an attempted decryption with a slightly incorrect key. UACI is similar, but it accounts for the amount of difference between two pixels in the same location. UACI is typically just used for measuring plain text sensitivity, and not general parameter sensitivity as well like NPCR. For 8 bit grey images, like the ones used in this paper, the expected values of NPCR and UACI for secure encryption techniques are 0.9961 and 0.3346 respectively [37]. NPCR and UACI are calculated from Eq 10.

$$\begin{array}{c} NPCR=\frac{\sum (I_{1}!=I_{2})}{H*W} \end{array}$$ (10a)

$$\begin{array}{c} UACI=\frac{\sum |I_{1}-I_{2}|}{H*W*255} \end{array}$$ (10b)

The NPCR and UACI values in Table 6 show ideal sensitivity to the plain image. What those values indicate specifically is the sensitivity of the cryptosystem to changes in all 5 hash parameters at the same time, as a result of a single bit change in the plain image. This can obscure if the system is insufficiently sensitive to any one parameter from the set of hash parameters. Table 7 includes the NPCR for small individual changes to all parameters for both encryption and decryption. The NPCR for a change to the hash iteration count was the weakest, and should not be considered secure.

**Table 7:** NPCR values for the rabbit image upon increasing a single parameter value by 1.

	Set Encryption	Set Decryption	Hash Encryption	Hash Decryption
Iterations + 1	0.9915	0.9915	0.9202	0.9202
Sq. Size + 1	0.9935	0.9935	0.9961	0.9961
Overlap + 1	0.9934	0.9934	0.9599	0.9599
P + 1	0.9933	0.9933	0.9602	0.9602
Q + 1	0.9935	0.9935	0.9943	0.9943

## Conclusion

CO_2_ OACM allows for the encryption of non-square images and has much higher periods than regular ACM. Ghost periods for OACM can greatly decrease the security of certain keys by limiting the effective iteration space. The overall image period for OACM is typically quite large, but is considerably smaller compared to the maximal order provided by the Landau function. The proposed simple cryptosystem indicates that OACM can perform decently as a tool in both permutation and diffusion. The histograms, NPCR, UACI, and correlation coefficients generally indicated resistance of the proposed cryptosystem to differential and statistical attacks. However, the sensitivity of the cryptosystem towards certain diffusion parameters such as the iteration count was too low to be considered secure.

Future related work may involve extending OACM to 3D, using partitions which wrap around an image’s boundaries, and using the 2D rectangular transform instead of ACM. Developing encryption techniques based on the Landau series is also of interest for future work.
